# Tetanus Following Serum Treatment

**Published:** 1918-01

**Authors:** 


***Tetanus Following Serum Treatment.*** By A. LUMIÈRE. Trans. and Abs. from the *Annales de l'Institut Pasteur*, t. XXXI, N° 1, Jan. 1917, p. 19.

Of 54 cases of tetanus following preventive treatment by serum, the author noted that 15 were of a light form without trismus; 13 were characterized by a late or weak form of trismus; but in 26 cases trismus was immediate and of the same severity as in cases which had not been treated preventively.

The author concludes from his observations that serum prophylaxis is by no means sure; that the duration of immunity is uncertain; that the outbreak of late tetanus is due to excessive secretion of toxin in the wounded region, or to the liberation of dormant spores, as a result of surgical intervention or of a subsequent injury. The outbreak, in either case, is delayed until the immunity afforded by the serum is exhausted.

L. believes that most of the early cases that occur in spite of the preventive injection, may be avoided by the careful cleansing of the contaminated wound, by removing foreign bodies, and by frequently repeating the injection of serum. He believes that late tetanus may also be avoided in the majority of cases by an injection of serum before a secondary operation.

As a means of relieving the painful spasms of tetanus, L. recommends the injection of persulphate of soda as more sedative than sulphate of magnesia and other narcotics.

